# Association of MiR-126 with Soluble Mesothelin-Related Peptides, a Marker for Malignant Mesothelioma

**DOI:** 10.1371/journal.pone.0018232

**Published:** 2011-04-01

**Authors:** Lory Santarelli, Elisabetta Strafella, Sara Staffolani, Monica Amati, Monica Emanuelli, Davide Sartini, Valentina Pozzi, Damiano Carbonari, Massimo Bracci, Elettra Pignotti, Paola Mazzanti, Armando Sabbatini, Renzo Ranaldi, Stefano Gasparini, Jiri Neuzil, Marco Tomasetti

**Affiliations:** 1 Department of Molecular Pathology and Innovative Therapies, Polytechnic University of Marche, Ancona, Italy; 2 Department of Biochemistry, Biology and Genetics, Polytechnic University of Marche, Ancona, Italy; 3 Department of Statistic Science, University of Bologna, Bologna, Italy; 4 Department of Medical Oncology, Hospital University of Ancona, Ancona, Italy; 5 Thoracic Surgery Unit, Hospital University of Ancona, Ancona, Italy; 6 Pathological Anatomy Unit, Hospital University of Ancona, Ancona, Italy; 7 Pneumology Unit, Hospital University of Ancona, Ancona, Italy; 8 Apoptosis Research Group, School of Medical Science and Griffith Health Institute, Griffith University, Southport, Queensland, Australia; 9 Molecular Therapy Group, Institute of Biotechnology, Academy of Sciences of the Czech Republic, Prague, Czech Republic; University of Windsor, Canada

## Abstract

**Background:**

Improved detection methods for diagnosis of malignant pleural mesothelioma (MPM) are essential for early and reliable detection as well as treatment. Since recent data point to abnormal levels of microRNAs (miRNAs) in tumors, we hypothesized that a profile of deregulated miRNAs may be a marker of MPM and that the levels of specific miRNAs may be used for monitoring its progress.

**Methods and Results:**

miRNAs isolated from fresh-frozen biopsies of MPM patients were tested for the expression of 88 types of miRNA involved in cancerogenesis. Most of the tested miRNAs were downregulated in the malignant tissues compared with the normal tissues. Of eight significantly downregulated, three miRNAs were assayed in cancerous tissue and adjacent non-cancerous tissue sample pairs collected from 27 formalin-fixed, paraffin-embedded MPM tissues by quantitative RT-PCR. Among the miRNAs tested, only miR-126 significantly remained downregulated in the malignant tissues. Furthermore, the performance of the selected miR-126 as biomarker was evaluated in serum samples of asbestos-exposed subjects and MPM patients and compared with controls. MiR-126 was not affected by asbestos exposure, whereas it was found strongly associated with VEGF serum levels. Levels of miR-126 in serum, and its levels in patients' serum in association with a specific marker of MPM, SMRPs, correlate with subjects at high risk to develop MPM.

**Conclusions and Significance:**

We propose miR-126, in association with SMRPs, as a marker for early detection of MPM. The identification of tumor biomarkers used alone or, in particular, in combination could greatly facilitate the surveillance procedure for cohorts of subjects exposed to asbestos.

## Introduction

Malignant pleural mesothelioma (MPM) is an aggressive tumor with poor prognosis, mostly linked to asbestos exposure [1]. Although the inhalation of asbestos fibers is a well known risk factor, the lack of clinical symptoms in the early stages of the disease as well as the lack of useful diagnostic markers makes early diagnosis very difficult [2]. Current challenge in the management of MPM includes the identification of sensitive and specific biomarkers that can be exploited to detect early neoplastic changes preferentially in a non-invasive manner thus facilitating the detection of MPM at an early stage, as well as for monitoring the progress of patients with MPM and their response to the treatments. A number of circulating tumour markers have been evaluated, but their sensitivity is low [3], [4]. Recently, soluble mesothelin-related peptides (SMRPs) have been suggested as promising biomarkers for MPM [5]. The level of SMRP of 1 nM was recommended as the best cut-off value to distinguish MPM patients from controls. However, this approach does not discriminate asbestos-exposed individuals from healthy controls. Thus, the levels of SMRPs in the blood have be proposed as a biomarker suitable for the diagnosis of existing MPM but not to identify high-risk subjects [6], [7]; neither is it useful as a screening tool.

MPM is characterized by a long latency period from the time of asbestos exposure to clinical diagnosis, suggesting that multiple somatic changes may be required for the tumorigenic conversion of normal mesothelial cells [8]. In this long promotion/propagation phase (typically >10 years), chromosomal rearrangement, aberrations and deletions as well as epigenetic changes have been proposed to occur.

It is known that epigenetic mechanisms are involved in the regulation of microRNAs (miRNAs) [9], [10], a class of naturally occurring small non-coding RNAs of 19–25 nucleotides in length. About 700 miRNAs have been identified in humans, with each miRNA affecting up to 200 target genes by blocking the translation of individual proteins [11]. These molecules are involved in the regulation of up to one-third of all human genes by promoting the degradation of target messenger RNA.

Aberrant expression of miRNAs has been shown to contribute to the pathogenesis of several human diseases [12]–[14] including cancer [15]–[17], and may serve as a valuable diagnostic or prognostic marker for a variety of pathologies. Therefore, the identification of a specific miRNA profile may be utilized for better identification of cancer types [18], [19]. We hypothesize that a profile of deregulated miRNAs may be used for the detection of MPM and that the levels of expression of specific miRNA species could help monitoring the disease development.

In this study, the miRNA profile associated with the development of MPM was evaluated by quantitative reverse transcriptase-polymerase chain reaction (qRT-PCR) analysis of biopsies freshly collected from patients with MPM and from healthy subjects used as controls. Certain deregulated miRNA species were selected and subjected to further analysis in a larger series of samples. Among the miRNAs analyzed, the miR-126 was found to be significantly downexpressed in malignant tissues. Next, we tested the applicability of the selected miR-126 as a circulating biomarker for early detection of MPM and risk-disease prediction. Thus, the miR-126 levels were evaluated in serum samples of asbestos-exposed subjects, defined as high-risk population to develop the disease, and MPM patients, correlated with the level of the angiogenic factor VEGF and SMRPs, and compared with healthy subjects. Our results indicate that the miR-126 expression, in particular in combination with SMRPs, may be used as a marker to help diagnose of the neoplastic disease and could greatly facilitate the surveillance procedure for cohorts of subjects exposed to asbestos.

## Results

### MiRNA expression profile distinguishes MPM from normal mesothelium

**Figure 1:**
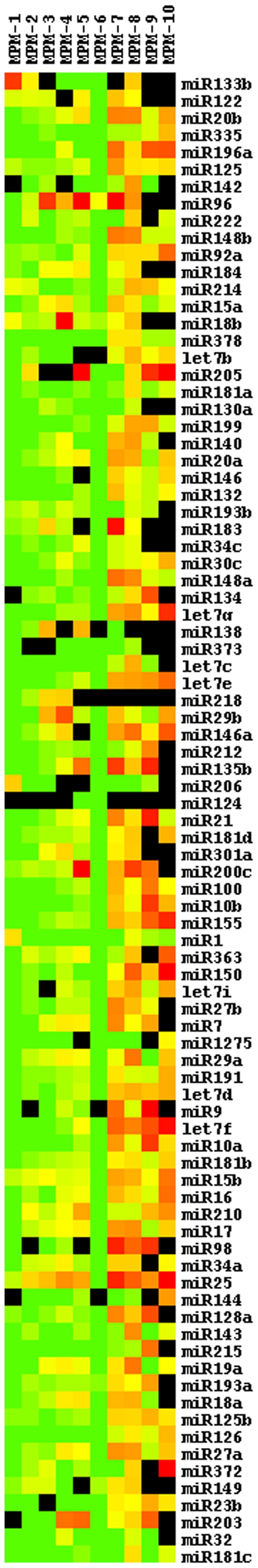
Hierarchical cluster analysis of miRNAs. miRNA expression of 10 MPM tissues is shown with respect to the pooled miRNAs from 5 normal tissues. MiRNAs were considered differentially expressed if their levels were increased or decreased by more than 2-fold. Relative normalized expression for each miRNA is represented by color intensity (green, downregulation; yellow, no change in expression; red, increased expression; black, miRNA not detected).

To determine the miRNA species differently expressed in the MPM tissue compared with the normal mesothelial tissue, we used a customized miRNA PCR array with 88 human miRNAs that are known to play a role in cancer. By comparing miRNAs from freshly collected MPM biopsies with pooled miRNAs from normal controls, a miRNA ‘signature’ was obtained. Most of the miRNAs were downregulated in the malignant tissue compared to the healthy control samples (Fig. 1). The most significantly downregulated miRNA species were miR-335 (fold change −17.8±1.9, p<0.009), miR-130a (fold change −9.3±3.3, p<0.047), miR193b (fold change −5.2±1.1, p<0.012), miR-30c (fold change −6.8±1.1, p<0.02), miR-212 (fold change −10.7±1.9, p<0.018), miR-126 (fold change −18.0±1.7, p<0.036), miR-32 (fold change −76.6±2.4, p<0.039), and miR-181c (fold change −14.3±2.4, p<0.046). Correlations were found between the levels of individual miRNA species, while no correlation was detected between miRNAs and the tumor stage (data not shown).

**Figure 2:**
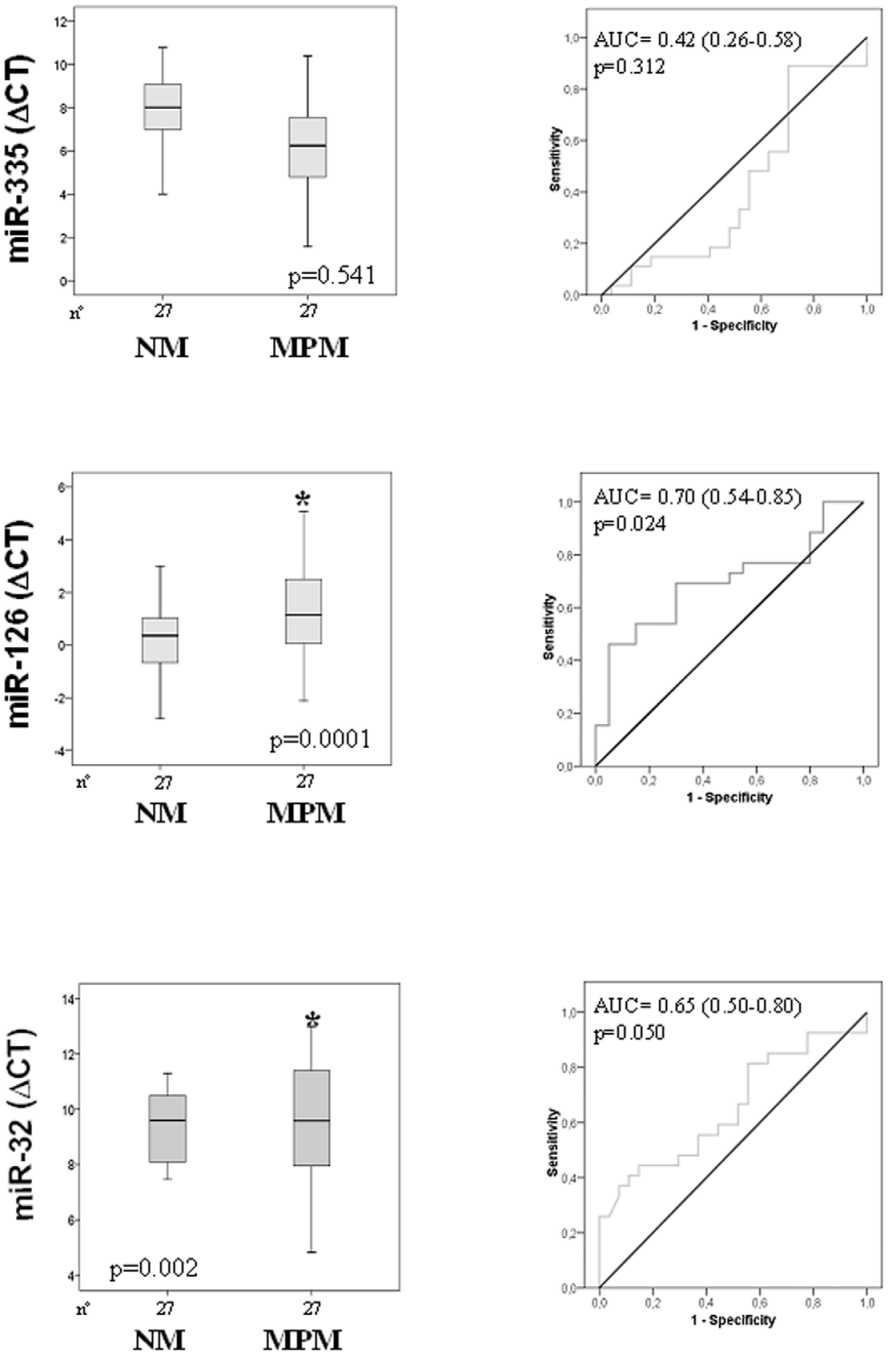
Box plot and ROC curves of miR-335, miR-126 and miR-32 expression levels. Distribution of miR-335, miR-126 and miR-32 expression levels (ΔC_T_) in malignant pleural mesothelioma (MPM) and normal mesothelial (NM) tissue (left panels). The areas under the receiver operating curves (AUC) were determined for miR-335, miR-126 and miR-32, discriminating cancerous and non-malignant tissues. Differences with *p*<0.05 were considered statistically significant. *MPM *vs.* NM.

Three miRNA species most consistently downregulated, with fold change of >15, were selected for a larger-scale analysis using the paraffin-embedded tissue sections. We therefore analyzed the expression of miR-335, miR-126 and miR-32 in the pairs of the cancerous and the adjacent non-malignant tissue. The ROC curves were generated to analyze the diagnostic value of individual miRNA. When the number of samples was increased, miR-335 lost its significance to discriminate the pathological tissue (MPM group) from the normal, control tissue (NM group), accuracy to 42% (26–58%, p = 0.312). Therefore, only miR-126, accuracy to 70% (54–85%, p = 0.024), and, to a lower extent, miR-32, accuracy to 65% (50–80%, p = 0.050), were significantly downregulated in the MPM group compared to the NM group allowing to discriminate between cancerous and non-malignant tissues (Fig. 2).

**Figure 3:**
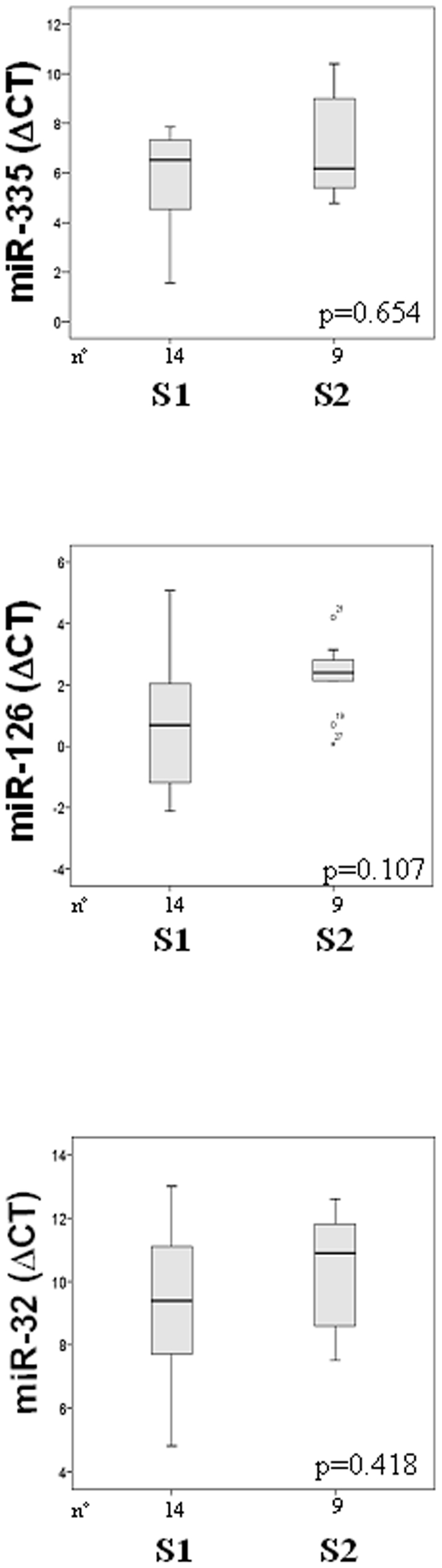
Box plot of miR-335, miR-126 and miR-32 expression levels according to tumor staging. Distribution of miR-335, miR-126 and miR-32 expression levels (ΔC_T_) in S1, MPMs staged S-Ia, S-Ib, S-II without lymph nodes and metastases involvement and S2, MPMs staged S-III, S-IV with lymph nodes and metastases involvement. *S1 *vs.* S2, *p*<0.05.

The MPM group was then divided into two sub-groups according to the stage of the disease. The MPM tumors staged S-Ia, S-Ib, or S-II without lymph nodes and metastases involvement were included in the S1 group, while the MPM tumors staged S-III or S-IV with lymph nodes and metastases were included in the S2 group. We then compared the expression of miR-335, miR-126 and miR-32 in these two groups. Although without reaching statistical significance, all miRNAs were more downregulated in the advanced S2 group (Fig. 3).

### Circulating miR-126 differentiates asbestos-exposed subjects from MPM patients and healthy controls

Recently, the expression profile of circulating miRNAs in the serum has been suggested as a potential biomarker for cancer detection [22]–[24]. It is widely accepted that asbestos inhalation is the predominant cause of MPM, with ∼80% of cases associated with documented asbestos exposure [1]. We hypothesized that the levels of specific miRNAs in the blood may be used to diagnose possible pathological changes associated with inhalation of asbestos fibres.

MiR-126, the most important marker in our previous screening (see above), was evaluated in a cohort of asbestos-exposed subjects defined as high-risk subjects, in MPM patients and in healthy controls.

**Figure 4:**
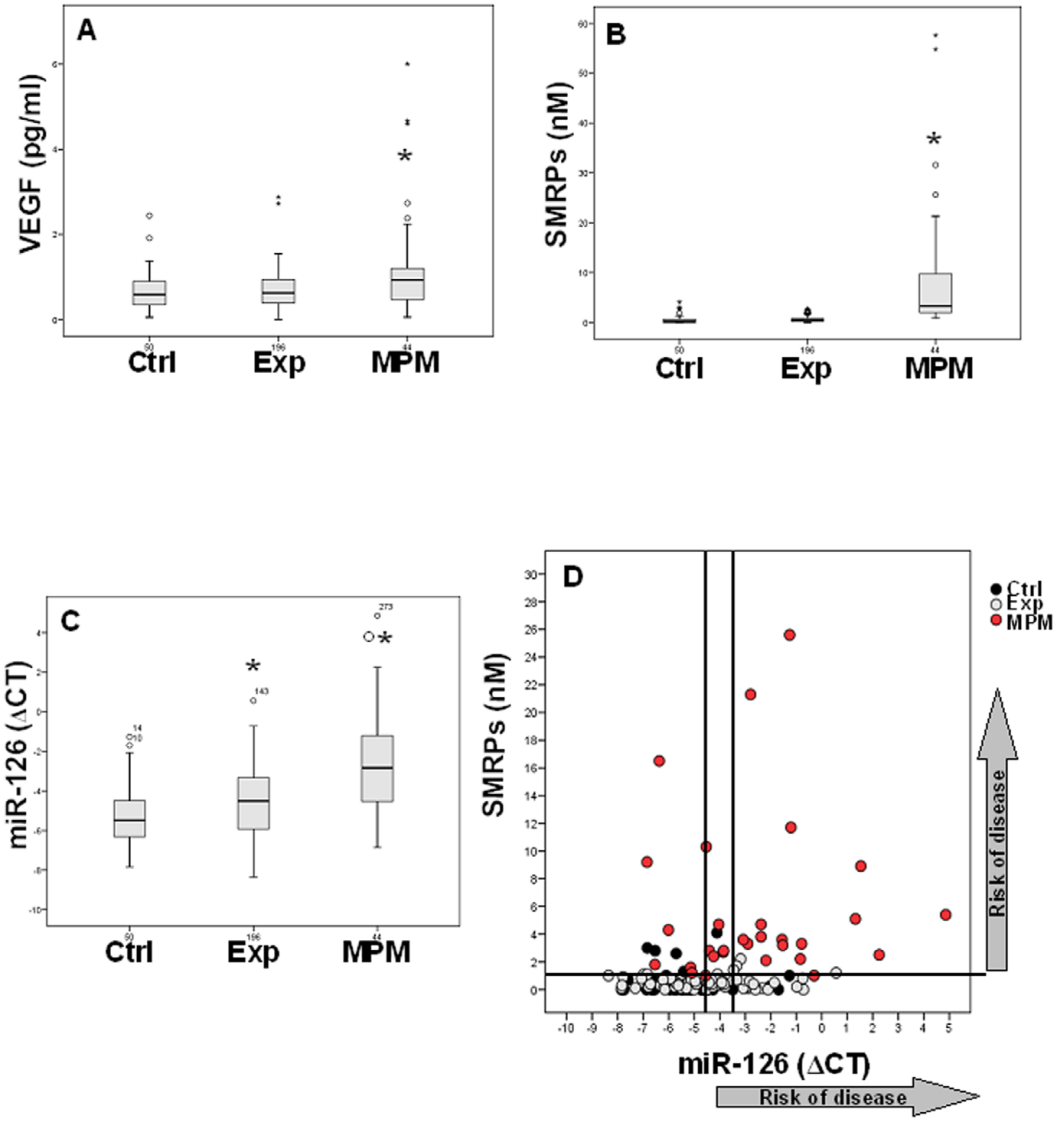
Box plots showing VEGF, SMRPs and miR-126 serum levels. Distribution of VEGF (A), SMRPs (B), miR-126 (C) levels and SMRPs-miR-126 association (D) in asbestos-exposed subjects (Exp); MPM patients and healthy controls (Ctrl) are shown. Based on the percentile analysis, a cut-off for SMRPs was determined to discriminate asbestos-exposed subjects and healthy controls from MPM patients. Two cut-offs were calculated for miR-126 to discriminate asbestos-exposed subjects from healthy controls (ΔC_T_ = −3.5) and from MPM patients (ΔC_T_ = −4.5) *Ctrl *vs.* Exp and MPM; Exp *vs.* MPM, *p*<0.05.

MiR-126 was correlated with the serum levels of the angiogenic factor VEGF and the SMRPs, a specific marker of MPM [5], [6]. Multivariate logistic regression analysis was performed to estimate the influence of independent factors on the level of miR-126. Among the various factors, such as asbestos-related diseases (fibrosis and pleural plaques), duration of asbestos exposure, cumulative fibre doses and markers of the disease (VEGF and SMRPs), only VEGF levels were found to strongly correlate with miR-126 (R = 0.659, *p* = 0.02). As shown in Fig. 4A,B, MPM patients showed higher serum VEGF and SMRP levels relative to the asbestos-exposed subjects and controls. On the other hand, miR-126 can significantly differentiate the high-risk individuals from the healthy controls and cancer group (Fig. 4C). Using ROC analysis, cut-off values of miR-126 were determined to discriminate asbestos-exposed subjects from controls (ΔC_T_ = −3.5; sensitivity 60% and specificity 74%) and from MPM patients (ΔC_T_ = −4.5; sensitivity 73% and specificity 74%).

To evaluate whether a combination of individual markers may increase the predictive value for early detection of MPM, SMRP levels, that can distinguish MPM patients from asbestos-exposed subjects and controls with a cut-off of 1 nM, have been found to correlate with serum miR-126 (Fig. 4D). The probability of the risk to develop the disease was higher with decreasing expression of miR-126, when correlated with increasing levels of SMRPs.

## Discussion

The prognosis of MPM patients is dismal despite current therapeutic modalities that include surgery, chemotherapy, and radiation of the thoracic drainage site [2], [25], [26]. In the early stage, surgery may offer a chance for prolonged survival, but patients need to be carefully selected, since less then 10% of the patients are eligible for this therapeutic option [27]. In advanced stages, chemotherapy with novel antifolates combined with cisplatin offers a rather small, albeit significant survival advantage [28]. Early and accurate diagnosis is important for appropriate therapeutic intervention, which may result in prolonged survival of MPM patients or, in the ideal case, their complete recovery.

Focus has been on finding tumor markers that can be used in association with radiography for MPM detection [29]. Several rather promising approaches have been suggested. For example, patients with MPM show increased serum levels of the MPM-specific peptide mesothelin and related peptides [5], [30], [31]. We have recently identified the combination of SMRPs and the level of expression of the vascular endothelial growth factor (VEGF) together with 8-hydroxy-2′-deoxyguanosine (a marker of oxidative stress) as a potential indicator of early and advanced MPM [6]. Although these reports are encouraging, we decided to explore a relatively different avenue to MPM diagnosis, based on the use of miRNA profiling.

Accumulating reports strongly indicate the potential diagnostic applications of miRNA in human cancers, also suggesting their possible use in therapeutic applications [19], [32], [33]. Therefore, miRNA expression profiles can be utilized to discriminate normal from malignant tissue, to identify the tissue origin in poorly differentiated tumors, or to distinguish cancers of unknown origin as well as their sub-types.

To identify a specific miRNA signature, we first analyzed human miRNAs with a potential role in malignant tissue freshly obtained from MPM patients, which was compared with corresponding samples of normal human mesothelium. This approach revealed differences in the expression profile of miRNAs in MPM samples and in the controls. We found that most miRNA species were expressed at lower levels in the MPM samples compared to the controls (*cf* Fig. 1). This result is in agreement with several studies that reported an overall downregulation of miRNAs in tumors compared to the corresponding normal tissue [34], [35].

An miRNA profile was previously identified by analyzing 17 biopsies freshly collected from MM patients for 723 human and 76 viral miRNAs [36]. Twelve miRNAs were highly expressed, whereas nine were found to be downregulated. More recently, Busacca and colleagues [37] evaluated miRNA expression profile in cultured mesothelioma cells. The significantly deregulated miRNAs were then further assessed by qRT-PCR and subsequently analyzed in 24 MM specimens, representative of three tumor histotypes (epithelioid, biphasic, and sarcomatoid). A pattern of deregulated miRNAs was found in these samples. Although well carried out, these two studies reported different profiles of miRNA expression, suggesting that both the selection of the samples and the applied methodological approaches could have affected the results. Gee and colleagues have recently suggested that miRNA analysis can be used to distinguish MM from lung carcinomas [38], which gives the potential application of miRNA profiling yet another level of importance.

In this study, the miRNA profile was determined using biopsies collected from MPM patients before the diagnosis and, therefore, the enrolled patients did not receive any adjuvant chemotherapy or radiation therapy that could affect expression of the individual miRNAs. Also, the qRT-PCR array we used allowed quantification of miRNA expression. Using this approach, we identified eight significantly downregulated miRNAs. Of these, three most consistently deregulated miRNAs were analyzed in 27 cancerous and adjacent non-malignant tissue sample pairs, resulting in the identification of miR-126 as promising markers that may be potentially utilized to distinguish cancerous and normal tissue. More specifically, we observed low levels of miR-126 in MPM samples, with their expression independent of tumour staging (*cf* Fig. 2,3).

The expression of miR-126 has been recently found to be low in human lung cancer cells. More specifically, miR-126 has a binding site in the 3′-untranslated region (3′-UTR) of the VEGF-A mRNA, and its upregulation resulted in decreased expression of VEGF-A. These results suggest a tumor suppressor function of miR-126 in the context of lung cancer [39], which is strongly dependent on the production of angiogenic factors. VEGF, which is secreted by tumor cells and is essential for tumour vascularization [40], is predicted to be a target for a variety of miRNA species [41], including miR-126 [39]. Collectively, miRNAs in cancer cells are likely to contribute to the regulation of tumor angiogenesis by affecting the paracrine signalling between cancer cells and endothelial cells of the vasculature.

One of the major challenges in MM is the identification of biomarkers for early detection of the disease, which can be routinely measured in surrogates. Recently, circulating miRNAs have been shown as promising biomarkers for detection of human cancers [42]. Here, we estimated the risk of MPM in an asbestos-exposed population via assessment of serum miR-126 in relation to asbestos exposure parameters, the angiogenic mediator VEGF and the tumor marker SMRPs. Multivariate logistic regression analysis revealed that miR-126 was not affected by asbestos exposure, whereas it was found strongly associated with VEGF levels. Low expression of miR-126 was correlated with high levels of VEGF (data not shown). High VEGF levels and SMRPs were found in the serum of MPM patients compared with asbestos-exposed subjects and healthy controls (*cf* Fig. 4A, B). As previously reported, SMRP levels can distinguish MPM patients from both asbestos-exposed subjects and controls with an estimated cut-off of 1 nM. Thus, the level of SMRPs have been proposed as a biomarker suitable for the diagnosis of existing MPM but not to predict the disease [6]. Conversely, miR-126 levels can significantly differentiate the high-risk individuals from healthy controls and the cancer group (*cf* Fig. 4C). Using ROC analysis, we calculated the cut-offs for clinical significance, resulting in dot plots of the combination of markers that were used to stratify the studied population. When combined with SMRPs, miR-126 indicates a better performance for the discrimination of subjects with high-risk to develop tumors, suggesting a potential diagnostic indicator for patients in the early stages of MPM (*cf* Fig. 4D).

In this study, we have identified miRNAs whose expression differs in the MPM tissue when compared to the corresponding healthy tissue. Of the various differentially expressed miRNAs, miR-126 was found to be significantly downregulated in the malignant tissue. Further, expression of miR-126 can be easily evaluated in the serum, and its level in association with a specific marker of MPM, SMRPs, can be used to identify subjects with high risk to develop the disease. The identification of tumor biomarkers used alone or, in particular, in combination could greatly facilitate the surveillance procedure for cohorts of subjects exposed to asbestos, a relatively common phenomenon in different areas of industrialized countries.

## Materials and Methods

### Ethics statement

All subjects filled a questionnaire including their informed consent. The study was carried out according to the Helsinki Declaration and the samples were processed under approval of the written consent statement by Ethical Committee of the University Hospital of Marche, Italy.

### Specimens

To obtain biopsy specimens, 22 subjects (aged 69.8±10.1 years; 20 males, 2 females) who underwent thoracoscopy or thoracotomy for suspected MPM were enrolled. The collected tissue was divided into two parts; one was immediately suspended in the RNALater solution (Ambion, Austin, TX, USA) and stored at −80°C until RNA extraction. The other tissue portion was used for histological examination by the Pathological Anatomy Unit of the Hospital University of Ancona, Italy. According to the diagnosis, the individuals were classified as subjects with MPM (the MPM group) and as healthy subjects with normal mesothelium (the NM group). The MPM group included tissue with clear signs of the pathology (n = 10), while the NM group included non-malignant tissue (n = 5). The exclusion criteria were the presence or suspicion of any infectious disease and other malignancies. Tumors were classified as epithelioid, sarcomatoid, biphasic, and the tumor stage evaluated based on the recommendation by the International Mesothelioma Interest Group (IMIG) [20]. The demographic and pathological characteristics of the subjects are summarized in Table 1.

**Table 1:** Demographic and pathological characteristics of individual subjects.

Biopsies	Age(years)	Sex(M/F)	Smoking(yes/ex/no)	Histotype(EP/BI/SA)	Stage
MPM-1	68	M	ex	EP	S-Ib
MPM-2	63	M	no	EP	S-Ia
MPM-3	66	M	no	EP	S-Ia
MPM-4	75	M	yes	EP	S-IV
MPM-5	81	F	yes	EP	S-III
MPM-6	70	M	ex	EP	S-Ia
MPM-7	75	M	ex	EP	S-III
MPM-8	66	M	no	EP	S-III
MPM-9	83	M	no	SA	-
MPM-10	77	M	no	EP	-
NM-1	80	F	yes	-	-
NM-2	58	M	yes	-	-
NM-3	83	M	no	-	-
NM-4	60	M	no	-	-
NM-5	61	M	no	-	-

EP, epithelioid; SA, sarcomatoid; S-Ia (any T1a); S-Ib (any T1b); S-II (any T2); S-III (any T3, any N); S-IV (any T4, any N, any M).

Formalin-fixed, paraffin-embedded (FFPE) tissue of the subjects affected by MPM (n = 27) was collected from the Archive of the Pathological Anatomy Unit of the Hospital University of Ancona, Italy. The FFPE samples were cut into 5 µm sections and stored at room temperature until analysis. The adjacent non-cancerous tissue was used as a control. The clinical data were obtained retrospectively and included information on the gender, age, histology, neoadjuvant chemoradiation and therapy administration (before surgery), smoking status and the pathologic staging. The MPM sub-types were as follows: 23 epithelioid, 1 sarcomatoid and 3 biphasic. The demographic and pathological characteristics of the subjects are summarized in Table 2. The patients were not treated with any adjuvant chemotherapy or radiation therapy.

**Table 2:** Demographic and pathological characteristics of MPM patients.

FFPE tissues	Age(years)	Sex(M/F)	Smoking(yes/ex/no)	Histotype(EP/BI/SA)	Stage
**MPM-1**	70	M	no	EP	S-IV
**MPM-2**	66	M	yes	EP	S-III
**MPM-3**	71	M	yes	EP	S-III
**MPM-4**	66	M	ex	EP	S-III
**MPM-5**	72	M	ex	EP	S-I
**MPM-6**	59	M	ex	EP	S-III
**MPM-7**	62	M	no	BI	S-I
**MPM-8**	80	M	no	EP	S-II
**MPM-9**	57	F	no	SA	S-I
**MPM-10**	78	M	ex	EP	S-III
**MPM-11**	70	M	yes	EP	S-III
**MPM-12**	75	M	yes	EP	S-III
**MPM-13**	75	M	no	EP	S-I
**MPM-14**	69	F	yes	EP	S-II
**MPM-15**	74	M	ex	EP	S-II
**MPM-16**	74	M	no	EP	S-I
**MPM-17**	68	M	no	EP	S-I
**MPM-18**	63	M	yes	EP	-
**MPM-19**	70	F	no	EP	-
**MPM-20**	45	M	no	EP	-
**MPM-21**	67	M	ex	EP	S-I
**MPM-22**	70	M	no	BI	S-I
**MPM-23**	75	M	yes	EP	S-I
**MPM-24**	44	M	no	EP	S-I
**MPM-25**	77	M	no	EP	-
**MPM-26**	78	M	ex	BI	S-III
**MPM-27**	73	M	ex	EP	S-I

EP, epithelioid; SA, sarcomatoid; BI, Biphasic; S-I (any T1a, any T1b); S-II (any T2); S-III (any T3, any N); S-IV (any T4, any N, any M).

### Study population

#### Asbestos-exposed subjects

From November 2004 to April 2010, 196 subjects (mean age 60.9±9.6 years, 188 males, 8 females) with a history of asbestos exposure were enrolled at the Institute of Occupational Medicine, Polytechnic University of Marche, Ancona, Italy. The participants were interviewed by trained personnel and answered a detailed questionnaire on duration of asbestos exposure, smoking and occupational tasks. Each subject underwent lung function analysis, chest radiography, and high-resolution computed tomography. A ‘fiber-year’ exposure metric was calculated for each subject, assigning to each person an arbitrary coefficient of ‘inhalated fibers (ff)’ indicating the occupational hazard. The ‘cumulative fibers’ (C_f_) are interpreted as the cumulative dose of asbestos fibers in the workplace of (ff/cm^3^)×yrs [21]. The subjects had been exposed to asbestos fibers on average for 23.3±10.7 years with a C_f_ of 28.8±50.4 (ff/cm^3^)×yrs. Smokers 87/196 (44%), ex-smokers 31/196 (16%) and non-smokers 78/196 (40%) were examined. Evidence of asbestos-related diseases (fibrosis and pleural plaques) was found in 56/196 (29%) subjects.

#### MPM patients

44 patients (mean age 63±10; 37 males, 7 females) diagnosed for MPM, were recruited, from November 2004 to January 2010, at the Oncology Clinic of the University Hospital of Ancona, Italy, and included smokers 18/44 (41%), ex-smokers 7/44 (16%) and non-smokers 19/44 (43%). Exclusion criteria were the presence or suspicion of any infectious disease, previous radical surgery, radiotherapy, as well as chemotherapy for MPM. Pathological diagnosis was performed on pleural biopsies obtained by thoracoscopy or thoracotomy. Tumors were classified as epithelial in 30, mixed in 8 and sarcomatoid in 6 patients, and the tumor stage was evaluated.

#### Healthy-controls

The control group consisted of 50 healthy subjects (mean age 68±8 years; 40 males, 10 females) recruited from November 2004 to January 2010, and included smokers 27/50 (54%), ex-smokers 4/50 (8%) and non-smokers 19/50 (38%). The subjects were undergoing screening radiography for chemoprevention at the Pneumology Clinic of the University Hospital of Ancona, Italy. None of them had ever been occupationally exposed to asbestos as documented by their occupational histories, and they presented with normal chest radiographs. Venous blood was collected from each subject at the time of clinical examination and serum prepared.

### Quantitative RT-PCR analysis

Total RNA was extracted from biopsies using Tri-Reagent (Sigma, St Louis, MO, USA) according to the manufacturer's instructions. MiRNAs were isolated from total RNA by the RT^2^ qPCR-grade miRNA isolation kit (SABiosciences, Frederick, MD, USA), and the cDNA synthesized using the RT^2^ miRNA First Strand kit (SABiosciences) according to the manufacturer's instructions. The expression of 88 miRNA species involved in human cancer development (array MAH-102A, SABiosciences) was assessed by qRT- PCR (Mastercycler EP Realplex, Eppendorf, Milano, Italy) using RT^2^ SYBR Green qPCR Master Mix (SABiosciences).

Total RNA from the FFPE tissue samples (10 µg) was obtained using the RecoverAll total nucleic acid isolation kit (Ambion, Austin, TX, USA) according to the manufacturer's instructions. Expression of selected miRNA species was quantified by qRT-PCR (Mastercycler EP Realplex) using the TaqMan MicroRNA Assay (Applied Biosystems, Foster City, CA, USA).

Circulating RNA was isolated by adding to 250 µl of serum an equal volume of Tri-Reagent BD (Sigma, St Louis, MO), the phase lock gel (Eppendorf) was used to improve RNA recovery. The miRNA isolation kit (SABiosciences) was used for miRNA purification. miRNAs were eluted in a final volume of 40 µl RNase free water and 4 µl were reverse-transcribed to cDNA using individual TaqMan MicroRNA Assay and the expression quantified by qRT-PCR.

To normalize the expression levels of target miRNA, the U6 small nuclear RNA was used as a control (housekeeping).

### Soluble mesothelin-related peptide (SMRP) assay

The level of SMRPs was assessed using a sandwich-type ELISA assay (Mesomark, Schering, Milano, Italy) according to the manufacture's instructions, and the results are expressed in nmol/l. Briefly, 100 µl of standard and plasma samples (1∶100 dilution) were added to each well of a 96-well microtitre plate coated with specific antibodies against SMRPs and incubated at room temperature for 60 min. After washing, the plate was incubated with a secondary HRP-conjugated antibody. The detection process included the addition of 100 µl of the TMB (3,3′,5,5′-Tetramethylbenzidine) substrate to each well and the absorbance was read at 405 nm using an ELISA plate reader (Sunrise, Tecan, Milano, Italy). Concentrations of SMRPs were extrapolated from the standard curve, and expressed in nM.

### Vascular endothelial growth factor (VEGF) assay

Human VEGF ELISA kit (EuroClone, Paignton, UK) was used according to the manufacturer's instructions to assess levels of the cytokine in serum samples. The results are expressed in pg/ml.

### Statistical analysis

The results were expressed as mean±S.D. of ΔC_T_ (C_T_ of miRNA - C_T_ of housekeeping) and high miRNA ΔC_T_ value corresponded to low miRNA expression. The fold changes in relative miRNA expression were calculated using the equation 2^−Δ(ΔCt)^. MiRNA species that were not detected in any of samples or with a C_T_ value >35 were excluded from the comparison. Differences with *p*<0.05 were considered statistically significant.

The miRNA species with at least a two-fold expression change between groups were considered differentially expressed. The cluster analysis was performed on the basis of the ΔΔC_T_ log values, and the resulting expression map was visualized with Treeview using the average-linkage clustering algorithms (Eisen Lab, Stanford University, CA, USA). The miRNA species with increased expression are indicated by red color, those with decreased expression are shown in green color. Yellow color indicates miRNAs whose expression was similar in the MPM and NM groups. Black color indicates miRNAs that were not detected.

Statistical significance of different expression between two groups was determined by means of the t-test and paired t-test. Multiple comparisons were determined by Analysis of Variance (ANOVA) followed by the Post-hoc LSD test. Correlations were performed according to Pearson. Receiver operating characteristics (ROC) curves were plotted to quantify the marker performance. The area under curve (AUC) indicates the average sensitivity of a marker over the entire ROC curve. The robustness of the models was evaluated using bootstrap techniques. The best statistical cut-offs were calculated by minimizing the distance between the point with sensitivity = 1 and specificity = 1 and the intercept on the ROC curve. Multivariate logistic regression model was used to estimate the influence of independent variables such as asbestos-related diseases (fibrosis and pleural plaques), duration of asbestos exposure, cumulative fibre doses, VEGF and SMRP levels on the selected miRNA.

The data were analyzed by the Statistical Package Social Sciences (version 15) software (SPSS, Chicago, IL, USA).

## References

[1] Hansen J, de Klerk NH, Musk AW, Hobbs MST (1998). Environmental exposure to crocidolite and mesothelioma.
Exposure-response relationships.. Am J Respir Crit Care Med.

[2] Tomasetti M, Amati M, Santarelli L, Alleva R, Neuzil J (2009). Malignant mesothelioma: Biology, diagnosis and new therapeutic
approaches.. Curr Mol Pharmacol.

[3] Romero S, Fernández C, Arriero JM, Espasa A, Candela A (1996). CEA, CA 15-3 and CYFRA 21-1 in serum and pleural fluid of
patients with pleural effusions.. Eur Respir J.

[4] Pass HI, Lott D, Lonardo F, Harbut M, Liu Z (2005). Asbestos exposure, pleural mesothelioma, and serum osteopontin
levels.. N Engl J Med.

[5] Scherpereel A, Grigoriu B, Conti M, Gey T, Gregorie M (2006). Soluble mesothelin-related peptides in the diagnosis of malignant
pleural mesothelioma.. Am J Respir Crit Care Med.

[6] Amati M, Tomasetti M, Scarrozzi M, Mariotti L, Alleva R (2008). Profiling tumour-associated markers for early detection of
malignant mesothelioma: an epidemiological study.. Cancer Epidemiol Biomarkers Prev.

[7] Luo L, Shi HZ, Liang QL, Jiang J, Qin SM (2010). Diagnostic value of soluble mesothelin-related peptides for
malignant mesothelioma: A meta-analysis.. Respir Med.

[8] Robinson BW, Musk AW, Lake RA (2005). Malignant mesothelioma.. Lancet.

[9] Rauhala HE, Jalava SE, Isotalo J, Bracken H, Lehmusvaara S (2010). miR-193b is an epigenetically regulated putative tumor suppressor
in prostate cancer.. Int J Cancer.

[10] Lodygin D, Tarasov V, Epanchintsev A, Berking C, Knyazeva T (2008). Inactivation of miR-34a by aberrant CpG methylation in multiple
types of cancer.. Cell Cycle.

[11] Krek A, Grün D, Poy MN, Wolf R, Rosenberg L (2005). Combinatorial microRNA target predictions.. Nat Genet.

[12] Heneghan HM, Miller N, Kerin MJ (2010). Role of microRNAs in obesity and the metabolic
syndrome.. Obes Rev.

[13] Zhang C (2009). MicroRNA-145 in vascular smooth muscle cell biology: A new
therapeutic target for vascular disease.. Cell Cycle.

[14] Barbato C, Ruberti F, Cogoni C (2009). Searching for MIND: microRNAs in neurodegenerative
diseases.. J Biomed Biotechnol.

[15] Shah PP, Hutchinson LE, Kakar SS (2009). Emerging role of microRNAs in diagnosis and treatment of various
diseases including ovarian cancer.. J Ovarian Res.

[16] Yanaihara N, Caplen N, Bowman E, Seike M, Kumamoto K (2006). Unique microRNA molecular profiles in lung cancer diagnosis and
prognosis.. Cancer Cell.

[17] Coppola V, de Maria R, Bonci D (2009). MicroRNAs and prostate cancer.. Endocr Relat Cancer.

[18] Esquela-Kerscher A, Slack FJ (2006). Oncomirs - microRNAs with a role in cancer.. Nat Rev Cancer.

[19] Calin GA, Croce CM (2009). MicroRNA signatures in human cancers.. Nat Rev Cancer.

[20] Rusch VW (1995). A proposed new international TNM staging system for malignant
pleural mesothelioma. From the International Mesothelioma Interest
Group.. Chest.

[21] Murphy RLH, Ferris BG, Burgess WA, Worcester J, Gaensler EA (1971). Effect of low concentration of asbestos: Clinical environmental,
radiologic and epidemiological observations in shipyard pipe coverers and
controls.. N Engl J Med.

[22] Mitchell PS, Parkin RK, Kroh EM, Fritz BR, Wyman SK (2008). Circulating microRNAs as stable blood-based markers for cancer
detection.. Proc Natl Acad Sci USA.

[23] Chen X, Ba Y, Ma L, Cai X, Yin Y (2008). Characterization of microRNAs in serum: a novel class of
biomarkers for diagnosis of cancer and other diseases.. Cell Res.

[24] Wang K, Zhang S, Marzolf B, Troisch P, Brightman A (2009). Circulating microRNAs, potential biomarkers for drug-induced
liver injury.. Proc Natl Acad Sci USA.

[25] Scagliotti GV, Selvaggi G (2007). Advances in diagnosis and treatment of malignant
mesothelioma.. Oncol Rev.

[26] Waller DA (2003). The role of surgery in diagnosis and treatment of malignant
pleural mesothelioma.. Curr Opin Oncol.

[27] Fennell DA, Gaudino G, O'Byrne KJ, Mutti L, van Meerbeeck J (2008). Advances in the systemic therapy of malignant pleural
mesothelioma.. Nat Clin Pract Oncol.

[28] Robinson BW, Lake RA (2005a). Advances in malignant mesothelioma.. N Engl J Med.

[29] Pass HI, Carbone M (2009). Current status of screening for malignant pleural
mesothelioma.. Semin Thorac Cardiovasc Surg.

[30] Robinson BW, Creaney J, Lake R, Nowak A, Musk AW (2003). Mesothelin-family proteins and diagnosis of
mesothelioma.. Lancet.

[31] Grigoriu BD, Chahine B, Vachani A, Gey T, Conti M (2009). Kinetics of soluble mesothelin in patients with malignant pleural
mesothelioma during treatment.. Am J Respir Crit Care Med.

[32] Ellis P, Davies AM, Evans WK, Haynes AE, Lloyd NS (2006). Lung Cancer Disease Site Group of Cancer Care Ontario's
Program in Evidence-based Care. The use of chemotherapy in patients with
advanced malignant pleural mesothelioma: a systematic review and practice
guideline.. J Thorac Oncol.

[33] Lu J, Getz G, Miska EA, Alvarez-Saavedra E, Lamb J (2005). MicroRNA expression profiles classify human
cancers.. Nature.

[34] Blenkiron C, Miska EA (2007). miRNAs in cancer: approaches, aetiology, diagnostics and
therapy.. Hum Mol Genet.

[35] Ortholan C, Puissegur MP, Ilie M, Barbry P, Mari B (2009). MicroRNAs and lung cancer: new oncogenes and tumor suppressors,
new prognostic factors and potential therapeutic targets.. Curr Med Chem.

[36] Guled M, Lahti L, Lindholm PM, Salmenkivi K, Bagwan I (2009). CDKN2A, NF2, and JUN are dysregulated among other genes by miRNAs
in malignant mesothelioma - an miRNA microarray analysis.. Genes Chromosomes Cancer.

[37] Busacca S, Germano S, De Cecco L, Rinaldi M, Comoglio F (2010). MicroRNA signature of malignant mesothelioma with potential
diagnostic and prognostic implications.. Am J Respir Cell Mol Biol.

[38] Gee GV, Koestler DC, Christensen BC, Sugarbaker DJ, Ugolini D (2010). Downregulated MicroRNAs in the differential diagnosis of
malignant pleural mesothelioma.. Int J Cancer.

[39] Liu B, Peng XC, Zheng XL, Wang J, Qin YW (2009). MiR-126 restoration down-regulates VEGF and inhibits the growth
of lung cancer cell lines in vitro and in vivo.. Lung Cancer.

[40] Kim KJ, Li B, Winer J, Armanini M, Gillett N (1993). Inhibition of vascular endothelial growth factor-induced
angiogenesis suppresses tumour growth in vivo.. Nature.

[41] Hua Z, Lv Q, Ye W, Wong CK, Cai G (2006). MiRNA-directed regulation of VEGF and other angiogenic factors
under hypoxia.. PLOS One.

[42] Cortez MA, Calin GA (2009). MicroRNA identification in plasma and serum: a new tool to
diagnose and monitor diseases.. Expert Opin Biol Ther.

